# Screening of Factors for Assessing the Environmental and Economic Efficiency of Investment Projects in the Energy Sector

**DOI:** 10.3390/ijerph191811716

**Published:** 2022-09-16

**Authors:** Anzhelika Pirmamedovna Karaeva, Elena Romenovna Magaril, Andrey Vladimirovich Kiselev, Lucian-Ionel Cioca

**Affiliations:** 1 Department of Environmental Economics, Ural Federal University, Mira-Str., 19, 620002 Ekaterinburg, Russia; 2 Department of Industrial Engineering and Management, Lucian Blaga University of Sibiu, 550024 Sibiu, Romania

**Keywords:** efficiency assessment, eco-modernization, energy sector, fossil fuels

## Abstract

In view of the current agenda in the field of climate and environmental conservation, the requirements for environmental project appraisal are being tightened: the evaluation of environmental indicators of project implementation should be carried out on a par with indicators of its economic performance. Current approaches to the assessment of environmental and economic efficiency do not completely cover the negative environmental impacts of a project’s implementation, and this reduces the effectiveness of the evaluation. Therefore, it is necessary to develop a system of environmental indicators that will address the specifics of the industry. This is made possible on the basis of determining a list of key factors that should be included in the evaluation system. The purpose of this study is to determine the most significant factors for establishing a simple yet thorough assessment framework to evaluate the efficiency of energy investment projects. Research methodology includes an a priori ranking method and analysis of interrelations between factors. Based on the results obtained, the authors have formed a list of key factors that could become the basis of a future system of environmental indicators for the efficiency assessment of energy projects.

## 1. Introduction

The modern agenda in the field of climate and environmental conservation and the global energy transition to low-carbon production are tightening the requirements for investment projects and their management [1,2,3]. The circular economy (CE) principles, including the availability of an effective waste management system, secondary use of waste generated for energy production or processing, and rational resource consumption, also significantly influence the justification of new projects [4,5,6]. Consequently, environmental indicators have a role to play in the decision-making process on the implementation of an investment project. Current approaches for environmental and economic project evaluation are more focused on evaluation of their economic performance: environmental efficiency and the environmental impact of the project is estimated at the stage of cash flow modeling, when environmental costs and benefits from the implementation of the project and environmental protection measures are calculated [6]. In most cases, the environmental component of the project is considered for evaluating investment projects that could be conditionally classified into two groups: criteria focused on accounting estimates (PP, investment performance indicator), and criteria based on discounted estimates (NPV, PI, IRR, DPP).

Negative environmental impact within the considered approaches is estimated as the ecological costs of minimizing environmental impact or the payment amount for pollution. Both ecological costs and pollution charges could vary considerably depending on the ecological legislation of the country and the availability of environmental technologies. That fact might lead to a reduction in the objectivity of estimations of environmental performance and makes it difficult to compare alternative investment projects [6,7]. For instance, a small amount of fines for pollution, due to the lack of strict environmental legislation, will have a lesser impact on economic efficiency, even if the investment project demonstrates a low level of environmental efficiency. In this case, when selecting investment projects, the real environmental component is not fully taken into account in the decision-making process and is reduced to a relatively small amount of environmental payments that overlap with potential profit [7].

In addition, current approaches to the appraisal of environmental and economic efficiency do not consider the specifics of various sectors of the economy, which complicates the comparison between alternative investment projects and negatively affects the effectiveness of decisions taken at the stage of their selection [8,9,10]. It is therefore necessary to develop simplified approaches to consider the specifics of economic sectors in order to improve the effectiveness of environmental performance assessment and the possibility of visual comparison of projects by their environmental component [11].

Energy is a strategic sector of the economy, ensuring the sustainable social development and economic well-being of a country and its national security. Energy consumption in 2021 was 14,221 Mtoe worldwide [12], and it will keep growing in the foreseeable future. 

**Figure 1:**
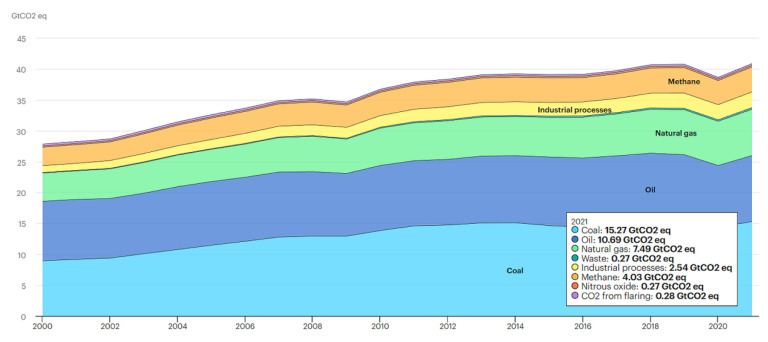
Energy related GHG emissions by source worldwide [13].

Almost 83% of consumed energy is obtained from fossil fuel. Oil is the major energy source, then in decreasing order, come natural gas and coal [13]. The generation of power based on fossil fuels has a number of critical impacts on environment, including the depletion of the ozone layer, greenhouse gas (GHG) emissions, global warming, air pollution, contamination of water bodies, soil contamination, the extinction of wildlife and rainforest loss [14]. For instance, more than 2/3 of global GHG emissions come from the energy sector. The total energy-related GHG emissions reached 40.8 Gt of CO_2_ equivalent (CO_2_ eq) in 2021 [15]. The global GHG emissions by source within the last decades is presented in Figure 1.

In the energy sector, carbon dioxide is the main contributor of the emission of GHGs, followed by nitrous oxide and methane [16], which have a much stronger GHG effect [17].

The strategic nature of the energy sector and its substantial environmental pressure mean that development and investment policies are of foremost significance. However, these issues are characterized by the following features:

- high capital intensity and long-term investment;
- regulated nature of operational and capital activities;
- limited external investment;
- organizational and technical complexity;
- high demand for qualified personnel.

For an accurate and comprehensive economic and environmental evaluation of the energy sector investment projects, the definition of the basic drivers which affect the matte is of a great importance.

There is a great variety of factors which influence the energy sector, all of which are affected by economic evolution, political background, agenda in the field of climate and environmental protection, technological state of the art, resource endowment, and the supply and demand trend in the market [18,19]. The way how these factors relate to assessing investment in energy projects is considered by multi-criteria decision-making methods (MCDMM). Gao et al. [20] highlighted four groups of factors—economy, environmental, social and risk, while Tao et al. [21] mentioned the following groups under MCDMM: economic benefits, technical benefits, social benefits and environmental benefits. Thus, environmental factors are necessarily part of the assessment procedure and are closely interrelated with other groups of factors.

The economic factors have significant impact on the evaluation process of projects in energy industries. Such indicators as the return on investment (ROI) and payback period are predominantly used for economic attractiveness of capital investment evaluation [22,23,24]. These values depend on a huge number of parameters that affect the investment costs, revenues and savings, and operational expenditures [24]. Capital costs for power plant construction have significant fluctuations depending on energy source and the technique used. Rocha et al. [25] give the following values for various energy sources, which refer to 2016: natural gas combined cycle 969–978 $/kW, advanced nuclear 5880–5945 $/kW, onshore wind 1686–1877 $/kW, photovoltaic 2277–2671 $/kW and coal 3493–5104 $/kW. Even the difference in the types of monofuel (e.g., coal) significantly affects the efficiency of the project, as it has different physical and economic characteristics: calorific value, cost per unit of production, composition of emissions through combustion, etc. Riansyah and Chalid [26] considered that generation of non-renewables, e.g., oil and coal, unexpectedly requires substantial CAPEX for exploration of the resources, which leads towards an increased risk regarding its availability over a long period.

The assessment of an investment project from cradle to grave reveals the majority of the factors affecting an investment project in the energy sector. It is worth saying that improvements in the technological process can provide significant savings. Szafranko [27] points out that investment into energy and resource efficiency decreases the negative environmental impact, reduces natural resources consumption, minimizes harmful emissions to the world around us, and creates possibilities to fulfill international obligations; these factors can be expressed in terms of performance indicators.

Gajdzik and Sroka [28] examined resource productivity and intensity as a crucial element of a company’s management, and highlighted this as an important factor for evaluating investment projects. The key objective for sustainable development is to increase the efficiency of the resources while decreasing the intensity of its consumption. The nature of a sustainable business model in terms of the circular economy concept lies in the understanding that resources are limited, particularly when speaking about energy, soil, water, spare parts and raw materials.

The increase of energy efficiency in the industrial and power sectors has become one of the key targets of energy policies in most of the world [29]. Yingjian et al. [30] consider the assessment of energy efficiency as a mandatory part of an energy investment project evaluation as it is directly connected with natural resource consumption and technological efficiency of energy production. A project could be considered effective if it uses less energy to ensure the same level of energy supply for buildings or technological processes in energy production [31,32].

Becchetti et al. [33] presented the Green Investment Financial Tool Approach (GIFTA) to provide indicators for measuring the environmental efficiency for private and public investments tools. They mentioned the following drivers for the GIFTA framework: mitigation of and adaptation to climate change, conscious use and maintenance of water resources, transition towards principles of circular economy, pollution control and ecosystem recovery. A suitable indicator (or set of indicators) was selected to evaluate each driver in terms of investments.

In contrast to previously mentioned research, Riansyah and Chalid [26] also considered local infrastructure and access to land, tax incentives, transparency of local authorities and regional asset-generating commissioning plan (including incentives for renewable energies) as factors affecting the feasibility study of energy investment projects.

The literature review presents a number of different groups of factors which affect the evaluation of energy investment projects’ efficiency. Among these factors both in and within functional areas (economic, environmental, technological and managerial), the volatile and multiple correlation was determined. At this point, the task of carrying out clear and sound assessment and having a common conclusion reached by various stakeholders seems to be a complex objective.

However, most researches admit the importance of environmental factors and indicators in the framework of investment projects’ efficiency evaluation. Despite a large number of studies devoted to improving approaches to assessing the environmental and economic efficiency of investment projects, the development of simplified approaches that allow for quick management decisions remains an urgent task.

To improve performance and objectivity of the environmental and economic efficiency evaluation process, approaches adapted to industry specifics need to be developed [11]. The development of adapted approaches will both allow consideration of the specifics of investment projects in different economy sectors under conditions of the transition towards a circular economy, and offer a balanced system of indicators for thoughtful management decisions.

In this paper, the authors focus on determining the minimum set of factors sufficient to conduct a qualitative ecological and economic assessment of investment projects in the energy sector, considering the specifics of the industry and its impact on the environment. The objective of this research is to define the most significant factors for establishing a simple and at the same time thorough assessment framework to evaluate the efficiency of energy investment projects. As a result, the authors compiled a list of the minimum number of factors which enable further development of environmental indicators for an efficiency assessment.

## 2. Materials and Methods

The study included the following stages:

1. Formation of a list of factors for the appraisal of environmental and economic project efficiency that will consider the specifics of the energy industry.

2. Double screening of selected factors to determine the minimum sufficient set of key factors for the appraisal of environmental and economic project efficiency.

2.1. Conducting the first screening of factors by a priori ranking by qualified experts. Selection of the most significant factors based on the results of processing the received data.

2.2. Conducting a second screening aimed at identifying the relationships between the factors selected at the previous stage. Determination of a minimum sufficient set of the most significant factors for the formation of a system of environmental indicators for evaluation of the environmental and economic efficiency of energy investment projects.

3. Approbation of the proposed system of environmental indicators in the example of a regional energy project for the eco-modernization of an energy facility.

### 2.1. Formation of a List of Factors for the Appraisal of Environmental and Economic Project Efficiency That Considers the Specifics of the Energy Industry

Based on the review and analysis of the scientific literature conducted by the authors, and on discussions with experts in the field of energy and environmental protection, as well as the authors’ existing experience, 44 factors were identified and classified. All factors are adjusted to the specifics of the energy industry and can be potentially included in the procedure for the appraisal of the environmental and economic efficiency of energy projects. The factors were divided into 5 groups:

1. Resource intensity of energy production;
2. Environmental payments;
3. Management of the energy facility;
4. Environmental costs and cost of energy production;
5. The environmental impact of the energy facility (atmospheric air; water resources; soil and land resources; and production-related waste generation).

**Figure 2:**
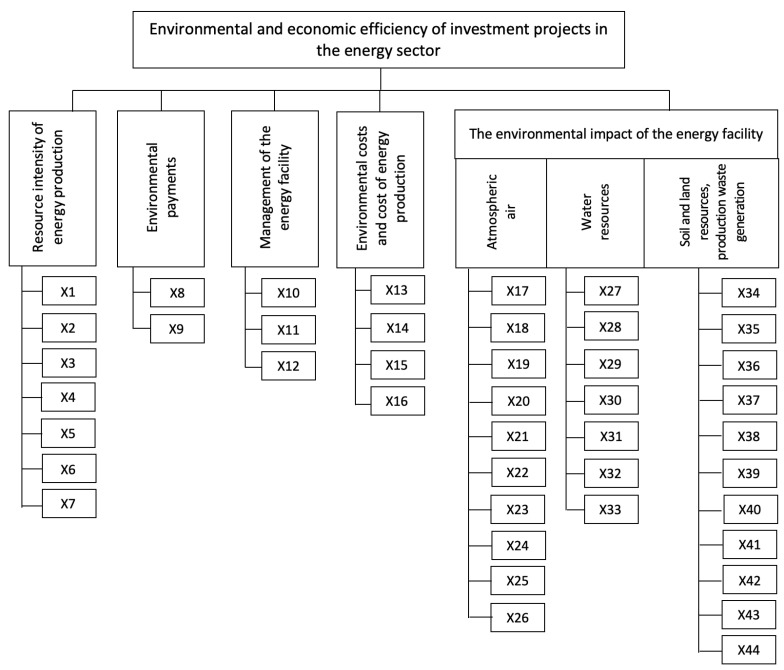
Classification of factors determining the environmental and economic efficiency of energy investment projects.

A factors tree is shown in Figure 2. Explanations of the factors’ codes from Figure 2 are presented in Table 1.

The authors believe that most of the factors are of limited significance in the environmental and economic assessment of investment projects due to their close relationship with other factors. According to the hypothesis of the study, those factors that are directly related to the type and amount of fuel used for energy production will have the greatest significance. The type of fuel has a direct impact on the resource efficiency of the project (consumption of fuel and water resources per unit of energy produced) and its environmental efficiency (the volume and composition of emissions of harmful substances and greenhouse gases, the volume and composition of discharges of harmful substances and the volume and composition of production-related waste).

### 2.2. Double Screening of Selected Factors to Determine the Minimum Sufficient Set of Key Factors for the Appraisal of Environmental and Economic Project Efficiency

For the first screening of factors, the authors used an a priori ranking method based on the individual assessment of factors by a group of experts with the required qualification in the study area.

To obtain more objective data, the authors compared the opinions of 10 experts who were divided into 2 groups: (1) ecologists working in the field of energy; (2) engineers working in the field of energy. Each group included 5 experts with at least 18 years of experience in the industry. Each group included:

- two experts holding major management positions in the industry;
- two research experts, working in universities or for the Academy of Sciences;
- one expert working at an energy company in scientific cooperation with universities.

The method allows the exclusion of the factors that have the least significant impact on the process under study. The advantages of the method are its simplicity and versatility. The disadvantages include the subjectivity of experts’ opinions and the influence of their qualifications on the final results. To obtain more objective data, the opinions of experts from several groups and different schools are compared. In this regard, the analysis was carried out with the invitation of two groups of experts: specialists in the field of energy and environmentalists.

The main stages of the a priori ranking method in relation to the purpose of the study were as follows:

1. Preparation of a questionnaire with a preliminary list of previously selected factors on the basis of the analysis of the factors affecting the environmental and economic performance of energy investment projects.

2. Formation of groups of qualified experts.

3. Instructing experts on filling out the questionnaire.

4. Individual assessment of the proposed factors by experts, with their placement in descending order of their influence on the energy project’s performance. The factor with the greatest influence is ranked in first place, the factor with the second greatest influence is ranked in second place, etc. If it is difficult to determine the significance of a factor in comparison with one or several other factors, then they are assigned consecutive places in a row, while indicating in the explanation that the factors having the corresponding codes have equal significance (related ranks).

5. Processing of the results of the expert survey:

5.1 Recalculation of related ranks into standardized ranks by dividing the sum of the places occupied by related ranks by their number;

5.2 Summarizing the survey results, and considering the recalculation of related ranks into a priori ranking tables (Appendix A);

5.3 Determination of the ranks sum of each factor;

5.4 Determination of the deviation of the ranks’ sum of each factor from the average sum of the ranks, $\Delta_{i}$;

5.5 Calculation of Kendall’s coefficient of concordance (*W*) (Equations (1)–(3)) for testing the hypothesis of the existence of consistency of expert opinions:

(1) $$W=\frac{S}{\frac{1}{12}m^{2}\left(k^{3}-k\right)-m\sum_{j}T_{j}}$$

(2) $$S=\sum_{i=1}^{k}\Delta_{i}$$

(3) $$T_{j}=\frac{1}{12}\underset{u}{\sum }\left(t_{u}^{3}-t_{u}\right)$$

where *m* is the number of experts, *k* is the number of factors, *u* is the number of groups formed by factors of the same rank in the *j*–th ranking, and *t_u_* is the number of identical ranks in the *u*–th group of the *j*-th ranking.

The concordance coefficient can vary from 0 to 1. If it differs significantly from zero, then we can assume that there is a certain agreement between the opinions of experts.

The significance of the concordance coefficient W is established using the Pearson’s chi-squared test. To do this, $\chi_{p}^{2}$ was found (Equation (4)):

(4) $$\chi_{p}^{2}=m\left(k-1\right)W$$

The calculated value of $\chi_{p}^{2}$ is compared to the table value of $\chi_{p}^{2}$ from the chi-square distribution table [44], found for the accepted significance level and the number of degrees of freedom *f = k − 1*. The hypothesis that the opinions of the experts are consistent is accepted if $\chi_{p}^{2}$ ≥ $\chi^{2}$.

5.6 Construction of a priori ranking diagram showing the distribution of factors by the sum of ranks;

5.7 Selection of the most significant factors.

One of the ways to identify the main factors is to compare the ranks of a given factor with their average values for all factors. The most significant factors are those whose sum of ranks does not exceed the average sum of ranks.

After the initial selection of factors by the a priori ranking method, the authors analyzed the interrelationships of factors in order to determine the minimum set of key factors having quantitative expression for the energy project’s environmental and economic evaluation.

Based on the results of the screening, a bubble diagram is constructed, with the designation of the main relationships between the factors; then, a list of key quantitative factors is formed as the basis of the system of indicators for the environmental and economic evaluation of energy investment projects.

### 2.3. Approbation of the Proposed System of Environmental Indicators in the Example of a Regional Energy Project for the Eco-Modernization of an Energy Facility

In order to test the results obtained, the authors carried out calculations of the specific performance indicators of the regional energy facility X (CHP), situated in the Sverdlovsk region, Russia, before and after implementation of the investment project on eco-modernization. The purpose of the considered energy project is the transition from a coal type of CHP to a gas–oil type. Prior to the start of the project, CHP X used coal as the main fuel type (Chelyabinsk brown coal) that led to the entry of significant amounts of harmful substances into the atmosphere. The implementation period of the energy project was 2 years. The project involved the complete eliminating of the old coal infrastructure. The continuous energy supply to the locality in which the CHP is situated should be provided in parallel. Table 2 shows the main performance indicators of the energy facility before and after implementation of the energy project.

## 3. Results

### 3.1. Key Factors for Environmental and Economic Evaluation of Energy Investment Projects

The expert analysis demonstrated a sufficient degree of consistency of the opinions of the interviewed experts: the concordance coefficient (*W*) is 0.55 which indicates that there is some consent between the opinions of the respondents. Testing of the hypothesis of non-randomness of experts’ agreement showed that with a 5% significance level and the number of degrees of freedom (*k −* 1) = 43, the calculated value of the Pearson criterion (234.86) is greater than the table one χ^2^ (59.3) which confirms the hypothesis of consistency of experts’ opinion and allows the use of the data obtained for further research.

In order to identify the most significant factors, the authors resorted to comparing the ranks of a given factor with their average value for all factors: those factors whose sum of ranks does not exceed the average sum of ranks are considered the most significant. The average sum of the ranks for 44 factors was 224.59.

**Figure 3:**
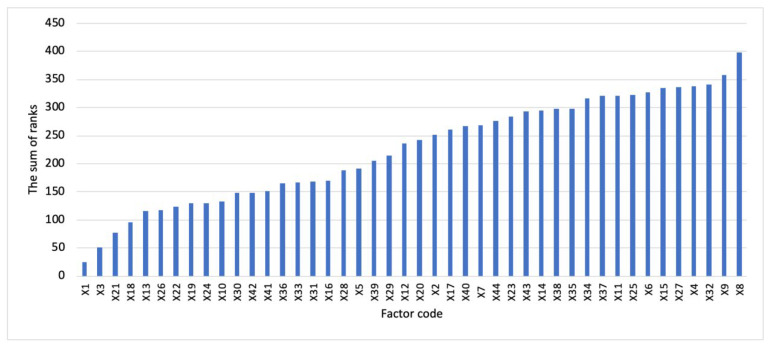
The diagram of a priori ranking of factors.

The processing of the results of the expert survey allowed the authors to determine the 20 most significant factors for the environmental and economic evaluation of energy projects. The results of a priori ranking of factors are presented in Table 3 and Figure 3.

It is necessary to analyze the relationship of quantitative and qualitative factors selected by experts in order to improve the system of appraisal of the environmental and economic efficiency of energy investment projects and to form a list of sufficient factors. Drawing up a list of sufficient factors will allow the determination of the key indicators that will form the basis of the future system for the environmental and economic efficiency evaluation of energy projects.

The type of fuel used (X1) has the smallest sum of ranks, thus being the most significant factor affecting the specific fuel consumption (X3) [34], the specific water consumption (X5) [46], the composition and structure of toxic emissions (X19) [41], the composition and structure of discharges of pollutants into water bodies (X29) [42,45], specific production-related waste generation (X39) and their hazard class (X41) [44]. In view of the different costs of fuels, it also influences the cost of energy production, for example, the average cost of 1 Btu of coal in 2021 in the United States averaged $1.98, compared to $4.98 for 1 Btu of natural gas [47].

The cost of energy production (X16) directly depends on the specific fuel consumption, as well as being associated with specific emissions of greenhouse gases (X18), toxic substances (X21), specific oxygen consumption (X24), specific wastewater discharges (X28) and production-related waste generation (X39). The factor of specific oxygen consumption (X24) correlates with the specific emissions of greenhouse gases (X18) and toxic substances (X21): the higher the volume of specific emissions from fuel combustion, the higher the oxygen consumption. Thus, this factor can be excluded from the assessment. Specific fuel consumption may indirectly affect the indicator of specific water consumption, but the determining factors of the efficiency of water resource use are the energy production technologies used at the energy facility and the type of fuel used [48].

It is advisable to include indicators of specific consumption of fuel and water resources, specific emissions of greenhouse gases and toxic substances, and specific wastewater discharge in the proposed system of indicators. These make it possible to evaluate comprehensively the resource efficiency of energy production and the negative impact of the facility on atmospheric air and water resources.

The factor of specific water consumption (X5) has a direct effect on cost of the energy production (X16), and on the specific wastewater discharges per unit of produced energy capacity (X28). Water consumption during energy production can reduce the cost of a unit of produced energy and the level of negative impact of an energy facility on water resources: the more rational the usage of water and the higher the quality of wastewater treatment from harmful impurities, the lower the specific volume of wastewater discharges and toxic substances contained in them. Emission treatment technologies in this case play a primary role.

Experts attributed the composition and structure of emissions of toxic substances (X 19), the toxicity of emission components (X22) and the toxicity of discharge components (X31) to significant factors, while factor X19 and factor X22 correlate with each other. The toxicity of emissions and discharges is characterized by the maximum permissible concentrations of pollutants established by sanitary and hygienic standards [49].

These quantitative factors could be used for the environmental and economic appraisal of energy projects as additional information about their negative effects on atmospheric air and water resources [50,51,52,53].

Specific formation of production waste (X39) affects the cost of energy production (X16) and the specific soil and land resource pollution per unit of produced energy capacity (X36). However, in terms of the environmental and economic appraisal, factor X42 “The specific volume of residual waste per unit of energy capacity produced” is more informative. It objectively assesses the efficiency of the waste management system at the energy facility: a low volume of residual waste is linked to the transfer of most of the production waste for recycling, reuse or disposal by specialized enterprises.

The hazard class of production waste (X41) also influences the factors X16 and X39: the lower the waste class, the lower the amount of costs and payments for processing and/or disposal of hazardous production-related waste. The hazard class of waste is an important component of assessing the impact of an energy facility on soil and land resources, but it does not allow for quantifying the scale of the impact. In this regard, factor X41 may be an additional, but optional, factor in the environmental and economic appraisal of energy investment projects.

The specific volume of residual waste per unit of produced energy capacity (X42) is primarily affected by the factor relating to the availability of an environmental management system (EMS) at the energy facility [37]. The presence of EMS implies the introduction of an efficient waste management system at the energy facility that contributes to an increase in the proportion of waste sent for recycling for secondary use in own or third-party production or sent to the companies responsible for the regional waste management.

The share of “green” investments in the total amount of investments (X13) allows us to evaluate the degree of environmental friendliness of the project in the context of investments: the higher the share of investments in environmental protection measures, the more likely the project will meet all relevant requirements in the field of environmental protection. Therefore, factor X13 can determine factors X10 (presence of EMS), X26 “Compliance of emission purification technologies with the best available technologies” and X33 “Compliance of discharge purification technologies with the best available techniques”. Factor X10 affects factors X26, X33, as well as X42 (as mentioned above, the presence of EMS implies the functioning of a waste management system at an energy company facility).

Compliance of emission purification technologies with the best available techniques (X26) directly affects the composition and structure of emissions of toxic substances (X19) and contributes to their reduction (X18). Compared to older systems, modern sewage treatment plants more effectively clean emissions from toxic gaseous substances and soot, thereby reducing the anthropogenic load [54,55,56].

A similar pattern is observed with respect to factors X33 “Compliance of waste treatment technologies with the best available techniques”, X30 “Specific volume of discharges of pollutants per unit of produced energy capacity” and X31 “Toxicity of discharge components”.

Factors X26 and X33 can have an impact on the cost of energy production (X16). For instance, according to the ecological legislation in Russia, in addition to benefitting from reduced fees for negative environmental impact, enterprises using best available techniques receive state support in the form of tax benefits and benefits for reducing negative impact on the environment in accordance with environmental legislation [49].

Since the factors X10, X13, X26, X33 are qualitative and interrelated with other factors having quantitative expression, they can be excluded from further consideration. Quantitative factors allow the assessment of change in the level of impact in dynamics (for example, before/after project implementation) and give an idea of the overall efficiency of the use of certain technologies in the framework of the project’s implementation [55].

The cost of energy production (X16) depends on most of the factors under consideration and is characterized by high economic significance. Its inclusion in the list of indicators for environmental and economic appraisal may be of a recommendatory nature: the cost structure is considered in more detail at the stage of forming the cash flows of an investment project to calculate its economic efficiency indicators.

**Figure 4:**
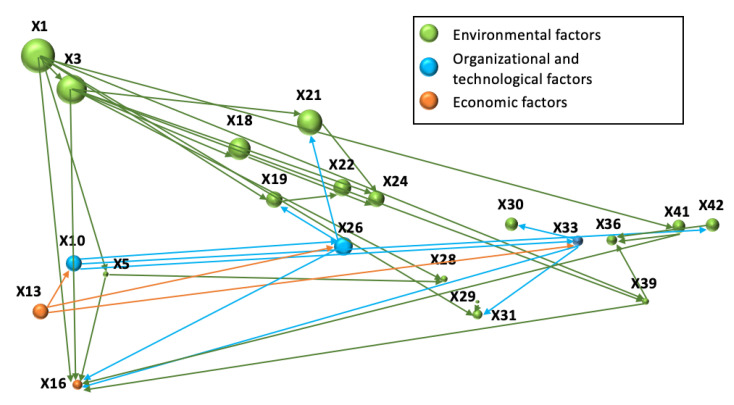
Interrelations between factors affecting the environmental and economic appraisal of energy projects.

A graphical representation of the factors’ relationship is presented in Figure 4.

The authors have compiled a list of key factors that make it possible to form a system of specific indicators for environmental and economic appraisal of energy investment:

- specific fuel consumption for energy production per unit of produced energy capacity;
- specific water consumption for energy production per unit of produced energy capacity;
- specific volume of toxic substance emissions per unit of produced energy capacity;
- specific volume of greenhouse gas emissions per unit of produced energy capacity;
- specific volume of discharges of pollutants per unit of produced energy capacity;
- specific volume of residual waste per unit of produced energy capacity;
- specific wastewater discharges per unit of produced energy capacity.

### 3.2. Case-Study

According to the available data on CHP X, the following indicators were calculated:

- specific fuel consumption for energy production per unit of produced energy capacity;
- specific water consumption for energy production per unit of produced energy capacity;
- specific volume of toxic substance emissions per unit of produced energy capacity;
- specific volume of greenhouse gas emissions per unit of produced energy capacity;
- specific volume of residual waste per unit of produced energy capacity.

To calculate the specific mass of emissions of toxic substances, it is necessary to estimate the reduced mass (*G*) considering the relative toxicity of the emission components (Equation (5)):

(5) $$G=\overset{n}{\underset{i=1}{\sum }}(G_{i}\cdot k_{i})$$

where *Gi* is the actual mass of the *i*-th pollutant entering the atmospheric air during the reporting period, tons; and *k_i_*, is the coefficient of relative environmental hazard of the *i*-th pollutant.

The calculation of the reduced mass of emissions is presented in Table 4.

Calculation of specific discharges of wastewater and toxic substances in this case is not required, due to the discharge of wastewater into the sewer, and not into a water body: in this case, discharges of toxic substances don’t affect the environmental state of water bodies, and in view of this, the volume of wastewater discharge and pollutants is not recorded.

Table 5 shows calculations of specific performance indicators of CHP X before and after the implementation of the investment project.

The specific fuel and water consumption decreased by 22.24% and 17.68%, respectively, while the total electricity generation increased by 7.37%. This indicates a substantial gain in efficiency of the resource consumption at the enterprise. In addition, there is a decrease in the negative impact on atmospheric air: the specific volume of toxic substance emissions and greenhouse gases decreased by 72.39% and 46.55%, respectively. The specific volume of residual production-related waste per unit of produced energy capacity also showed a significant decrease—by 50.64%.

The considered investment project is effective from an environmental point of view. The use of specific indicators selected as a result of the analysis of the interrelation of factors made it possible to compare the options “before project implementation” and “after project implementation”, greatly simplifying the evaluation procedure. Despite the lack of numerous data and the simplicity of calculations, the indicators are informative and quite comprehensively assess the change in the resource efficiency of an energy facility and the degree of its negative environmental impact.

Energy investment projects might differ significantly in economic, technological and organizational aspects, and consequently, the number of calculated indicators also might vary. For instance, some proposed environmental indicators may not be calculated in a number of cases: (i) if the project initially provides for a level of wastewater treatment sufficient to drain into the sewer; (ii) if the entire volume of waste is sent for processing to third-party organizations and/or is used for a second time at the enterprise and they are not considered in annual environmental reporting.

The list of key factors selected as a result of the double screening makes it possible to cover the areas of negative environmental impact of energy enterprises during the environmental and economic evaluation. In the majority of works devoted to the appraisal of the environmental efficiency of investment projects for energy enterprises, the most common indicators of assessment are the amount of fuel consumption and the amount of greenhouse gas emissions into the atmosphere [58]. Greater analysis of the environmental impact on atmospheric air, namely the composition, structure, volume and toxicity of emissions, is presented in [59,60,61,62,63], whereas only the gross volume of emissions of toxic substances is estimated. The structure of toxic emissions depends to a greater extent on the fuel used at the energy facility, and at the same time, its analysis at the stage of development of investment projects contributes to the correct choice of treatment facilities.

Various approaches to assessing energy facilities’ impact on water resources are presented in [36,38,63], but specific indicators that simplify the assessment procedure are not used by those authors.

The most informative characteristics are the specific consumption of fuel and water resources per unit of energy produced and the specific volume of residual production-related waste per unit of energy produced. These key figures could form a group of indicators for assessing the resource intensity of energy production. The further development of a group of integral indicators will allow evaluating the overall environmental and resource efficiency of a project as components of the environmental and economic appraisal.

In the development of the previously obtained results, the research conducted made it possible to identify key quantitative factors in energy projects’ environmental and economic appraisal that consider the specifics of the energy industry and enable a list of environmental indicators to be formed, the use of which will simplify its procedure. The proposed list of key factors, therefore, is the basis for further development of a methodology for the environmental and economic appraisal of energy projects.

The stated hypothesis of the research was confirmed.

## 4. Conclusions

According to the research, the key factors of energy projects’ environmental and economic evaluation were justified, considering the specifics of the energy industry’s effects on the environment. Thus, the hypothesis of the study is confirmed: the most significant factors are those that are directly related to the type and amount of fuel used for energy production. To improve approaches to the environmental and economic evaluation of energy projects, it is encouraged to apply these factors as a basis for developing a system of specific environmental indicators that will allow assessing the resource efficiency and the degree of projects’ impact on atmospheric air, water, soil and land resources. Proposed indicators could be calculated in physical and/or monetary units. This will considerably complement the economic justification of an energy investment project’s appraisal.

The use of a system of specific indicators will improve the evaluation quality, simplify its procedure and enable comparing alternative investment objects with each other. It could be used both to evaluate the efficiency of both new investment projects and existing energy enterprises already operating.

The proposed list of indicators is mostly applicable for traditional energy enterprises that produce energy using fossil fuels (coal, natural gas, oil). The evaluation factors and, consequently, indicators should be revised for nuclear power plants and renewable energy sources in order to consider the specifics of their operation.

## Figures and Tables

**Table 1:** Names of factors determining the environmental and economic efficiency of investment projects in the energy sector.

Factor Code	Name of the Factor	Reference
*1. Resource intensity of energy production*		
X1	Type of fuel used (natural gas, coal, fuel oil)	[9,34]
X2	Fuel consumption for energy production per year	[34]
X3	Specific fuel consumption for energy production per unit of produced energy capacity	[9]
X4	Water consumption for production needs per year	[35]
X5	Specific water consumption for energy production per unit of produced energy capacity	[35]
X6	Land occupation for the implementation of the investment project	[36]
X7	Land occupation per unit of produced energy capacity	[9,36]
*2. Environmental payments*		
X8	The amount of annual environmental payments (fee for emissions, fee for discharges, fee for waste disposal)	proposed by experts
X9	The amount of environmental payments (fee for emissions, fee for discharges, fee for waste disposal) per unit of produced energy capacity	proposed by experts
*3. Management of the energy facility*		
X10	Availability of an environmental management system at the energy facility	[37]
X11	Compliance of the energy facility management system with international standards	proposed by authors
X12	Availability of a waste management system at the energy facility	[38]
*4. Environmental costs and cost of energy production*		
X13	The share of “green” investments in the total amount of project investments	proposed by authors
X14	Availability of own energy raw materials for energy production in the region of the project implementation	proposed by experts
X15	Availability of the necessary transport infrastructure	proposed by experts
X16	Cost of energy production	[25]
*5. The environmental impact of the energy facility*		
*Atmospheric air*		
X17	Volume of greenhouse gas emissions per year	[39]
X18	Specific volume of greenhouse gas emissions per unit of produced energy capacity	proposed by authors
X19	Composition and structure of toxic substance emissions	[40]
X20	Volume of toxic substance emissions per year	[41]
X21	Specific volume of toxic substance emissions per unit of produced energy capacity	proposed by authors
X22	Toxicity of emission components	[41]
X23	Volume of oxygen consumption during fuel combustion per year	proposed by authors
X24	Specific oxygen consumption during fuel combustion per unit of produced energy capacity	proposed by authors
X25	Thermal pollution of the atmosphere	proposed by authors
X26	Compliance of emission purification technologies with the best available techniques	proposed by authors
*Water resources*		
X27	Volume of wastewater discharges per year	[42]
X28	Specific wastewater discharges per unit of produced energy capacity	proposed by authors
X29	Composition and structure of discharges of pollutants into water bodies	[42]
X30	Specific volume of discharges of pollutants per unit of produced energy capacity	proposed by authors
X31	Toxicity of discharge components	[42]
X32	Thermal pollution of the water bodies	proposed by authors
X33	Compliance of waste treatment technologies with the best available techniques	proposed by authors
*Soil and land resources, production-related waste generation*		
X34	The degree of change in the natural landscape of territories during the construction of an energy facility	[36]
X35	Volume of soil and land resource pollution	[36]
X36	Specific soil and land resource pollution per unit of produced energy capacity	[36]
X37	Thermal pollution of the soil	proposed by authors
X38	Production-related waste generation per year	[43]
X39	Specific production waste generation per unit of produced energy capacity	[9]
X40	Land occupation for storage of production-related waste	proposed by experts
X41	Hazard class of production-related waste	[43]
X42	Specific volume of residual waste per unit of produced energy capacity	[9]
X43	The volume of waste used as secondary resources in own production per unit of produced energy capacity	proposed by authors
X44	The volume of production-related waste sent for useful use to other enterprises per unit of produced energy capacity	proposed by authors

**Table 2:** Key indicators of the CHP X operation before and after investment project implementation.

Indicator	Before Project Implementation	After Project Implementation
Energy production, kWh	178,550	191,700
Total fuel consumption *, tons/tons of fuel equivalent including:	539,220/387,520	224,500/323,520
Natural gas, m^3^/tons/tons of oil equivalent	121,900/97,500/140,670	275,000/220,000/317,350
Fuel oil, tons/tons of oil equivalent	3700/5070	4500/6170
Coal, tons/tons of oil equivalent	438,000/241,780	0
Water consumption, m^3^	175,280	154,920
Residual production waste, tons	319,300	169,200
Total emissions of harmful substances, tons including	0.89	0.75
SO_2_, tons	0.39	0.02
NO_x_, tons	0.17	0.15
CO, tons	0.14	0.49
PM, tons	0.19	0.09
CO_2_ emissions, tons	933,668	535,769

* For converting tons and m^3^ to tons of fuel equivalent, authors used the following coefficient: natural gas = 1.154; fuel oil = 1.37; coal = 0.552 [45].

**Table 3:** The results of a priori ranking of factors for environmental and economic evaluation of energy investment projects.

Name of the Factor	Factor Code	The Sum of Ranks	
		Absolute Value	%
Type of fuel used (natural gas, coal, fuel oil)	X1	25.5	0.26
Specific fuel consumption for energy production per unit of produced energy capacity	X3	51	0.52
Specific volume of toxic substance emissions per unit of produced energy capacity	X21	77.5	0.78
Specific volume of greenhouse gas emissions per unit of produced energy capacity	X18	95.5	0.96
Share of “green” investments in the total amount of project investments	X13	116	1.17
Compliance of emission purification technologies with the best available techniques	X26	117	1.18
Toxicity of emission components	X22	123.5	1.25
Composition and structure of toxic substance emissions	X19	130.5	1.32
Specific oxygen consumption during fuel combustion per unit of produced energy capacity	X24	130.5	1.32
Availability of an environmental management system at the energy facility	X10	132.5	1.34
Specific volume of discharges of pollutants per unit of produced energy capacity	X30	148.5	1.50
Specific volume of residual waste per unit of produced energy capacity	X42	148.5	1.50
Hazard class of production-related waste	X41	151	1.53
Specific soil and land resource pollution per unit of produced energy capacity	X36	165.5	1.67
Compliance of waste treatment technologies with the best available techniques	X33	166	1.68
Toxicity of discharge components	X31	169	1.71
Cost of energy production	X16	170	1.72
Specific wastewater discharges per unit of produced energy capacity	X28	188	1.90
Specific water consumption for energy production per unit of produced energy capacity	X5	191.5	1.93
Specific production-related waste generation per unit of produced energy capacity	X39	206	2.08
Composition and structure of discharges of pollutants into water bodies	X29	215	2.17

**Table 4:** Reduced mass of harmful substance emissions before and after project implementation.

Name of Substance	k_i_ *	Before Project Implementation		After Project Implementation	
		tons	Reduced Mass, tons	tons	Reduced Mass, tons
SO_2_	20.00	0.39	7.80	0.02	0.40
NO_x_	16.50	0.17	2.81	0.15	2.48
CO	0.40	0.14	0.06	0.49	0.20
PM	2.70	0.19	0.51	0.09	0.24
Total	-	0.89	11.17	0.75	3.31

* Source: [57].

**Table 5:** Calculations of specific performance indicators of CHP X before and after project implementation.

Indicator	Before Project Implementation	After Project Implementation
Specific fuel consumption for energy production per unit of produced energy capacity	2.17	1.69
Specific volume of toxic substance emissions per unit of produced energy capacity (considering the relative toxicity of the emission components, Table 4)	0.06	0.02
Specific volume of greenhouse gas emissions per unit of produced energy capacity	5.23	2.79
Specific water consumption for energy production per unit of produced energy capacity	0.98	0.81
Specific volume of residual waste per unit of produced energy capacity	1.79	0.88

## Data Availability

Not applicable.

## Appendix A

**Table A1:** Results of a priori ranking of factors.

Group of Factors	Name of the Factor	Factor Code	Number of the Expert										The Sum of Ranks	
			1	2	3	4	5	6	7	8	9	10	Absolute Value	%
Resource intensity of energy production	Type of fuel used (natural gas, coal, fuel oil)	X1	1	1.5	8.5	6.5	3	1	1	1	1	1	25.5	0.26%
	Fuel consumption for energy production per year	X2	33	34	37.5	33.5	28.5	35	2	28	10	10	251.5	2.54%
	Specific fuel consumption for energy production per unit of produced energy capacity	X3	2	6	8.5	17.5	3	4	3	3	2	2	51	0.52%
	Water consumption for production needs per year	X4	34	39.5	37.5	33.5	28.5	36	42	29	28	30	338	3.41%
	Specific water consumption for energy production per unit of produced energy capacity	X5	3	16	8.5	26.5	17.5	17	41	18	24	20	191.5	1.93%
	Land occupation for the implementation of the investment project	X6	35	39.5	29	38.5	37.5	37	4	44	30	33	327.5	3.31%
	Land occupation per unit of produced energy capacity	X7	21.5	26.5	29	33.5	28.5	28	5	35	29	32	268	2.71%
Environmental payments	The amount of annual environmental payments (fee for emissions, fee for discharges, fee for waste disposal)	X8	37	42.5	40.5	43.5	44	38	44	33	39	36	397.5	4.02%
	The amount of environmental payments (fee for emissions, fee for discharges, fee for waste disposal) per unit of produced energy capacity	X9	36	34	40.5	43.5	37.5	20	43	34	34	35	357.5	3.61%
Management of the energy facility	The share of “green” investments in the total amount of project investments	X10	4	16	8.5	6.5	17.5	23	11	23	16	7	132.5	1.34%
	Availability of own energy raw materials for energy production in the region of project implementation	X11	43	42.5	29	33.5	41.5	24	10	22	42	34	321.5	3.25%
	Availability of the necessary transport infrastructure	X12	44	26.5	19	17.5	17.5	25	32	24	17	13	235.5	2.38%
Environmental costs and cost of energy production	The share of “green” investments in the total amount of project investments	X13	5	6	8.5	6.5	3	2	9	2	43	31	116	1.17%
	Availability of own energy raw materials for energy production in the region of project implementation	X14	6	16	43.5	41.5	41.5	26	7	32	38	43	294.5	2.97%
	Availability of the necessary transport infrastructure	X15	42	16	43.5	41.5	41.5	27	8	27	44	44	334.5	3.38%
	Cost of energy production	X16	7	16	29	33.5	17.5	3	6	4	33	21	170	1.72%
The environmental impact of the energy facility (atmospheric air)	Volume of greenhouse gas emissions per year	X17	21.5	44	29	26.5	28.5	34	18	30	7	23	261.5	2.64%
	Specific volume of greenhouse gas emissions per unit of produced energy capacity	X18	8	16	8.5	17.5	9.5	7	17	6	3	3	95.5	0.96%
	Composition and structure of toxic substance emissions	X19	23	6	40.5	17.5	9.5	5	13	5	6	5	130.5	1.32%
	Volume of toxic substance emissions per year	X20	24	34	29	26.5	28.5	29	16	25	8	22	242	2.44%
	Specific volume of toxic substance emissions per unit of produced energy capacity	X21	9	6	8.5	6.5	9.5	8	15	7	4	4	77.5	0.78%
	Toxicity of emission components	X22	10	1.5	29	6.5	9.5	6	14	36	5	6	123.5	1.25%
	Volume of oxygen consumption during fuel combustion per year	X23	25	39.5	29	26.5	28.5	39	20	26	26	24	283.5	2.86%
	Specific oxygen consumption during fuel combustion per unit of produced energy capacity	X24	11	16	8.5	17.5	9.5	9	19	8	23	9	130.5	1.32%
	Thermal pollution of the atmosphere	X25	38	26.5	19	38.5	28.5	40	21	37	37	37	322.5	3.26%
	Compliance of emission purification technologies with the best available techniques	X26	31	6	8.5	6.5	3	19	12	14	9	8	117	1.18%
The environmental impact of the energy facility (water resources)	Volume of wastewater discharges per year	X27	26	34	29	26.5	37.5	41	39	38	36	29	336	3.39%
	Specific wastewater discharges per unit of produced energy capacity	X28	12	26.5	8.5	6.5	28.5	18	34	17	21	16	188	1.90%
	Composition and structure of discharges of pollutants into water bodies	X29	27	16	29	17.5	17.5	10	35	13	22	28	215	2.17%
	Specific volume of discharges of pollutants per unit of produced energy capacity	X30	13	16	8.5	6.5	9.5	11	38	12	19	15	148.5	1.50%
	Toxicity of discharge components	X31	14	16	29	6.5	9.5	12	36	9	20	17	169	1.71%
	Thermal pollution of the water bodies	X32	40	34	40.5	38.5	28.5	21	40	21	40	38	341.5	3.45%
	Compliance of waste treatment technologies with the best available techniques	X33	32	6	8.5	6.5	3	22	33	19	18	18	166	1.68%
The environmental impact of the energy facility (soil and land resources, production-related waste generation)	The degree of change in the natural landscape of territories during the construction of an energy facility	X34	39	34	19	26.5	37.5	30	22	31	35	42	316	3.19%
	Volume of soil and land resource pollution	X35	28	34	29	38.5	28.5	42	24	20	27	27	298	3.01%
	Specific soil and land resource pollution per unit of produced energy capacity	X36	15	16	8.5	17.5	28.5	13	23	16	14	14	165.5	1.67%
	Thermal pollution of the soil	X37	41	16	19	26.5	17.5	43	37	41	41	39	321	3.24%
	Production-related waste generation per year	X38	29	39.5	19	33.5	41.5	44	26	15	25	25	297.5	3.01%
	Specific production-related waste generation per unit of produced energy capacity	X39	16	26.5	8.5	17.5	17.5	31	25	42	11	11	206	2.08%
	Land occupation for storage of production-related waste	X40	30	26.5	29	26.5	28.5	14	30	43	13	26	266.5	2.69%
	Hazard class of production-related waste	X41	17	6	29	6.5	9.5	15	27	10	12	19	151	1.53%
	Specific volume of residual waste per unit of produced energy capacity	X42	18	16	8.5	6.5	17.5	16	28	11	15	12	148.5	1.50%
	The volume of waste used as secondary resources in own production per unit of produced energy capacity	X43	19	26.5	29	17.5	28.5	32	29	39	32	40	292.5	2.95%
	The volume of production-related waste sent for useful use to other enterprises per unit of produced energy capacity	X44	20	26.5	8.5	17.5	28.5	33	31	40	31	41	277	2.80%
The sum of ranks		-	990	990	990	990	990	990	990	990	990	990	9900	100.00%
